# Three decades of microcystin-LR removal in aquatic environments: physical, chemical and biological strategies for environmental detoxification

**DOI:** 10.1098/rsos.260913

**Published:** 2026-09-09

**Authors:** Xin Zhong, Huixia Zhang, Ping Xie

**Affiliations:** ^1^ Longyan University, Longyan, People's Republic of China; ^2^ College of Life Sciences, Hebei University, Baoding, People's Republic of China; ^3^ Donghu Experimental Station of Lake Ecosystems, Institute of Hydrobiology, The Chinese Academy of Sciences, Wuhan, People's Republic of China

**Keywords:** microcystin-LR, physical adsorption, materials, aquatic environments

## Abstract

Microcystin-LR (MC-LR) contamination from cyanobacterial blooms poses a persistent threat to aquatic ecosystems and human health, necessitating effective removal technologies. This study used visualization analyses to examine global research on MC-LR removal in aquatic environments over the past three decades. A total of 978 publications from the Web of Science Core Collection, published from 1993 to August 2025, were analysed. Annual output increased slowly from 1993 to 2003, accelerated thereafter, and remained high after 2020, indicating that research activity entered a relatively stable phase following a period of rapid growth. *Water Research*, *Chemosphere*, *Chemical Engineering Journal*, *Journal of Hazardous Materials*, and *Environmental Science & Technology* were identified as leading journals, while China and the United States dominated research output and formed the central axis of international collaboration. Keyword and co-citation analyses showed that physical adsorption, photocatalysis, advanced oxidation processes and biodegradation are the main removal pathways. The field has diversified from early adsorption-assisted removal and conventional oxidation toward mechanism-driven and material-enabled strategies, while microbial degradation has remained an important parallel research direction. Findings suggest future progress should prioritize standardized evaluation of removal and detoxification efficiency, transformation-product toxicity, material regeneration, cost and scalability, thereby supporting reliable and practical cyanotoxin control.

## 1. Introduction

Global warming and pronounced eutrophication can intensify cyanobacterial blooms, thereby increasing cyanotoxin production [1,2]. Cyanobacteria produce potent toxins that can severely impact the human digestive and nervous systems [3]. Among these, MC-LR, the most prevalent microcystin (MC), is characterized by a cyclic structure with an added side chain and exhibits remarkable chemical stability, contributing to its role as a tumour promoter [4,5]. Mechanistically, MC-LR inhibits protein serine/threonine phosphatases 1 and 2A, leading to excessive phosphorylation of cytoskeletal proteins and thereby disrupting cellular structure and adhesion [6]. This disruption leads to cell death and may promote carcinogenesis [7]. In eutrophic freshwater ecosystems, MC-LR concentrations frequently exceed the World Health Organization's safety threshold of 1 µg l^−1^, posing significant risks to both ecosystems and human health [8,9]. Given these ecological and public health threats, research has increasingly focused on elucidating the transformation and degradation pathways of MC-LR in natural water bodies. Pioneering studies in the 1990s demonstrated the biodegradation and biotransformation of MCs in aquatic environments and provided early evidence for the enzymatic degradation pathway of MC-LR [10–13].

Over the past two decades, numerous innovative methods have been devised to remove MC-LR. In 2024, Shen *et al.* demonstrated that biochar-supported sulfide nanosized zero-valent iron (nZVI) and persulfate sustained-release agent (S-nZVI-BC/PSSA) effectively degrade MC-LR and Microcystis aeruginosa through free radical generation, offering a sustainable and efficient solution for eutrophic water treatment by converting MC-LR into CO_2_ and H_2_O [14]. In 2025, Ren *et al.* investigated nitrate-facilitated photodegradation of MC-LR in eutrophic waters, uncovering a novel degradation pathway and achieving complete removal of MC-LR under sunlight. These findings offer a sustainable, solar-driven solution for water treatment, with significant environmental relevance [15]. In 2026, Stoll *et al.* demonstrated that gold-decorated nickel metal–organic framework (Au/Ni-MOF) anodes were demonstrated to efficiently degrade MC-LR via electrochemical oxidation driven by hydroxyl radicals, achieving high removal efficiency with low energy consumption and showing strong potential for energy-efficient treatment of cyanotoxins in complex water systems [16].

However, previous research has mainly examined individual treatment technologies and their underlying mechanisms, while systematic analyses integrating publication trends, collaboration networks, thematic evolution and the intellectual structure of MC-LR removal research remain scarce. Although MC-LR is the most extensively studied MC congener in degradation research, previous reviews have generally addressed MC removal as a broad topic, while dedicated bibliometric analyses focusing specifically on publication trends, collaboration networks, and thematic evolution in MC-LR removal research remain scarce [17,18]. Visualization analysis, as a research approach based on mathematical and statistical methods, can systematically reveal the developmental trajectory and research hotspots of a given field through quantitative analysis of publication trends, collaboration networks among authors and institutions, keyword co-occurrence relationships and high-impact studies [19,20]. Therefore, it is necessary to conduct a systematic analysis of MC-LR-related research in order to comprehensively identify research hotspots, evolutionary trends and potential future directions, and to provide theoretical support for water environment management and risk control.

This study reveals the current research progress and technological applications of MC-LR removal in aquatic environments through visualization-based analysis. The purposes of this study are to: (i) analyse research trends, core journals and global collaboration patterns in MC-LR removal studies; (ii) identify the major countries, institutions, authors and co-cited references contributing to the development of this field; (iii) summarize the main removal pathways, including physical adsorption, photocatalysis, advanced oxidation processes and biodegradation, with a focus on material design, reaction mechanisms and integrated treatment systems; and (iv) discuss existing challenges and future perspectives for improving the efficiency, reliability and practical applicability of MC-LR removal technologies in cyanotoxin control.

## 2. Material and methods

### 2.1. Data source and search criteria

In this study, data were retrieved from the Web of Science Core Collection, a widely used database that indexes a large number of scientific publications each year [21,22]. The database provides information on publication output, research areas, authors, journals, citations and institutional affiliations. The search was completed on 25 August 2025, using the following query: ((‘Microcystin-LR’ OR ‘Cyanoginosin-LR’ OR ‘MCYST-LR’ OR ‘MC-LR’ OR ‘Microcystin-leucine arginine’) AND (degrad* OR decompos* OR remov* OR eliminat* OR treat* OR adsorb* OR sorption*)). Only articles and reviews were included, and after relevance screening, a final dataset of 978 records was retained (supplementary material, fig. S1). To improve the reliability of subsequent analyses, records were considered irrelevant and excluded if they did not address MC-LR removal, degradation, adsorption, transformation or treatment. Keyword abbreviations, spelling variants, synonymous terms and typographical variants were standardized [23,24]. Details of the keyword standardization procedures are provided in supplementary material, table S1.

### 2.2. Data visualization

Visualization was performed using VOSviewer (version 1.6.18; Leiden University, Netherlands), CiteSpace and Origin 2025. VOSviewer was used to construct and visualize co-authorship, institutional, national and keyword co-occurrence networks because of its ability to present large science mapping structures in an intuitive manner [25]. CiteSpace was applied to detect citation bursts, cluster co-citation networks and generate time-line views, thereby helping identify emerging topics and pivotal nodes in the field [26,27]. Origin 2025 was used to visualize annual publication trends.

In the generated science mapping networks, node size represents the importance or frequency of an item, such as an author, institution, country, keyword or cited reference, with larger nodes indicating greater influence [25,28]. Edge thickness reflects the strength of the relationship between two nodes, while the spatial distance between nodes indicates the degree of relatedness, with closer nodes representing stronger associations [26,29]. These tools provided an integrated framework for exploring the knowledge structure, collaboration patterns and research evolution of MC-LR removal studies. Notably, the AI tool was used solely to improve the manuscript's grammar, clarity and readability. We carefully reviewed and approved all AI-assisted revisions.

## 3. Results

### 3.1. Annual publication trends and leading journals

**Figure 1.**
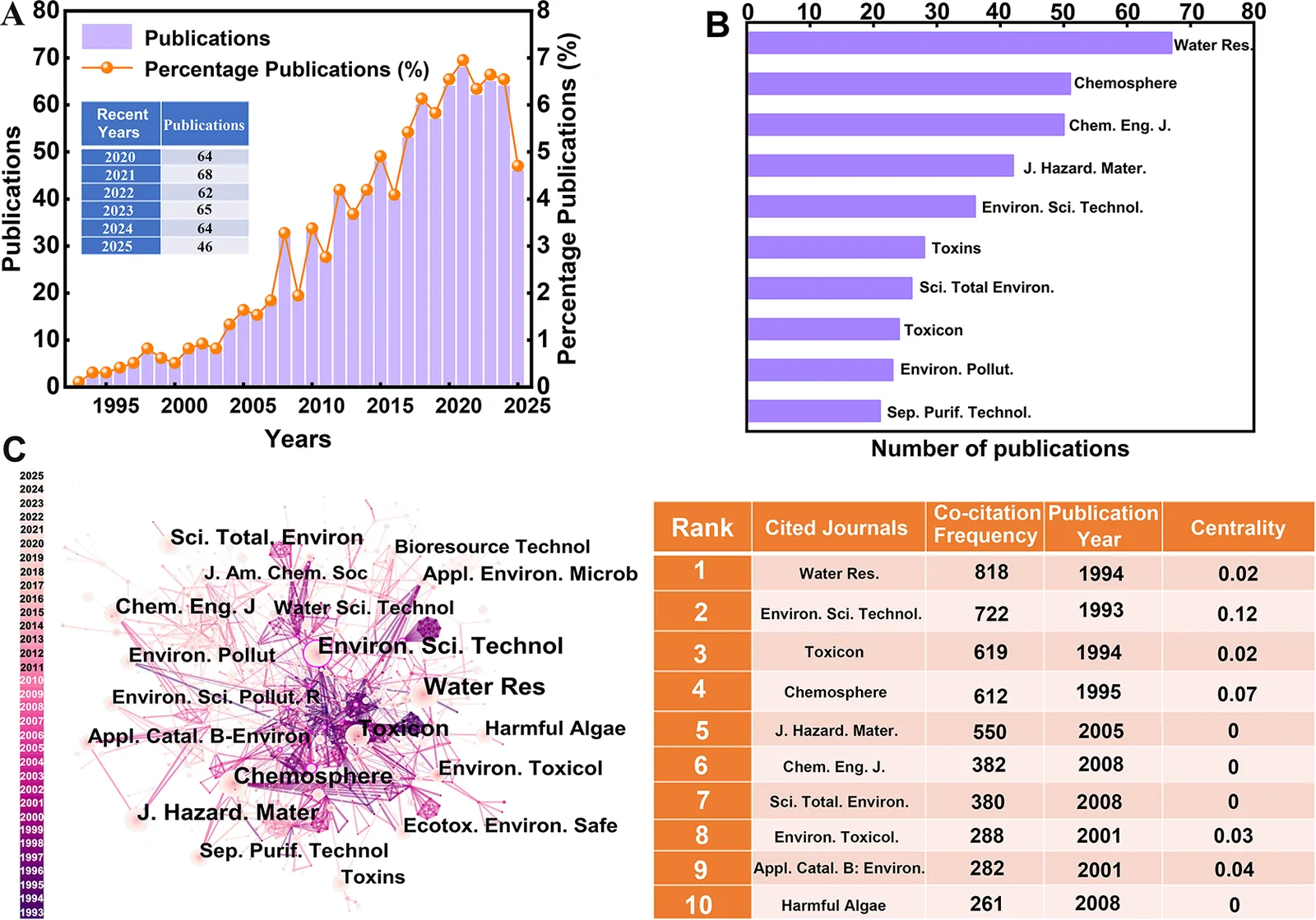
(A) Annual publication trends on MC-LR removal from 1993 to 2025. (B) Top 10 journals contributing to MC-LR removal research. (C) Journal co-citation network illustrating citation frequency by node size and annual citation trends by ring width.

As shown in figure 1A, research on MC-LR removal exhibited an overall upward trend over the study period, despite year-to-year fluctuations. During the early stage of development (1993–2003), the annual number of publications increased gradually from one to eight, laying the foundation for subsequent development. After 2003, publication output generally increased at a faster pace, marking a transition to sustained growth in this field. The number of papers peaked at 68 in 2021, and annual output remained above 60 from 2020 to 2024. As of August 2025, 46 publications had already been recorded. In total, 978 articles and reviews from 297 publication sources were included in the final dataset.

As shown in figure 1B, the 10 most productive journals are *Water Research, Chemosphere, Chemical Engineering Journal, Journal of Hazardous Materials, Environmental Science & Technology, Toxins, Science of the Total Environment, Toxicon, Environmental Pollution* and *Separation and Purification Technology*. Among them, *Water Research* ranks first with 67 papers (6.85%), followed by *Chemosphere* (51 papers, 5.21%) and *Chemical Engineering Journal* (50 papers, 5.11%). The top 10 journals accounted for 368 publications, representing 37.63% of the total output. This distribution indicates that MC-LR removal studies are primarily published in major environmental and engineering journals, with research addressing both fundamental mechanisms and applied technologies for water safety. To further assess journal influence within the analysed dataset, the average number of citations per included publication was analysed. *Environmental Science & Technology* had the highest average citations per publication, with 86.64, followed by *Water Research* with 69.79. *Toxicon* also showed a relatively high average of 57.83 citations per publication, indicating its substantial influence within the analysed MC-LR removal literature (supplementary material, table S2). Finally, the co-citation analysis, which examines how frequently journals are cited together by the same publications, identified *Water Research* (818 co-citations) and *Environmental Science & Technology* (722 co-citations) as major knowledge sources in MC-LR removal research (figure 1C; supplementary material, Text S1). *Environmental Science & Technology* exhibited the highest network centrality (0.12), highlighting its central role in shaping this research field.

### 3.2. The leading contributing countries, institutions and authors

**Figure 2.**
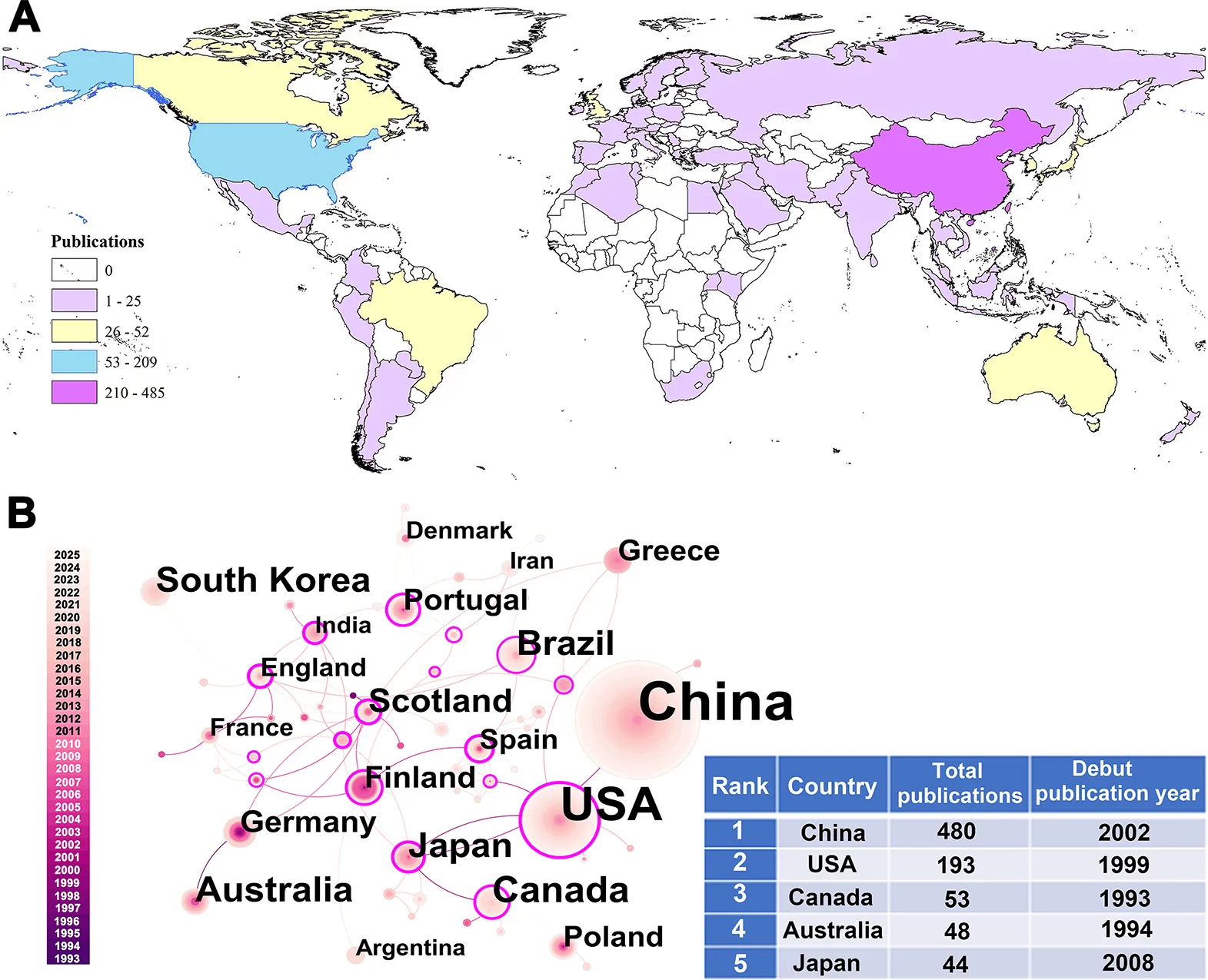
(A) Global distribution of MC-LR removal studies based on 978 publications from 1993 to August 2025. (B) National collaboration network, where node size and label denote publication volume (larger indicates more output), and the width of annual rings represents the number of papers published in each year.

An analysis of 978 publications on MC-LR removal research reveals a distinct global hierarchy, with China and the United States leading in scientific output (figure 2A). The top five countries—China (480 articles), the United States (193), Canada (53), Australia (48) and Japan (44)—account for 83.6% of total publications, underscoring the concentration of research activity within a few nations. To clarify the principal contributors and collaborative patterns, data from the Web of Science were analysed using CiteSpace and VOSviewer, with ‘country’ defined as the network node. In the resulting visualization, node size represents publication volume, and the radial width of each ring reflects annual output, with broader rings indicating greater productivity. The network comprises 65 national entities connected by 93 collaboration links, where line thickness denotes the strength of bilateral cooperation. As shown in figure 2B and supplementary material, fig. S2, China maintains extensive international collaboration, particularly with the United States, Japan and Australia. The prominent link between China and the United States highlights a strong bilateral research partnership, while the United States also engages actively with Canada and other countries, reflecting a broad collaborative reach. Beyond China and the United States, India, Brazil, Greece and Poland were also visible in the country collaboration network, indicating broader geographical participation in MC-LR removal research. Among the major contributors, Canada first entered this field in 1993, followed by Australia in 1994 and the United States in 1999, illustrating the temporal diffusion of MC-LR removal research across continents.

**Figure 3.**
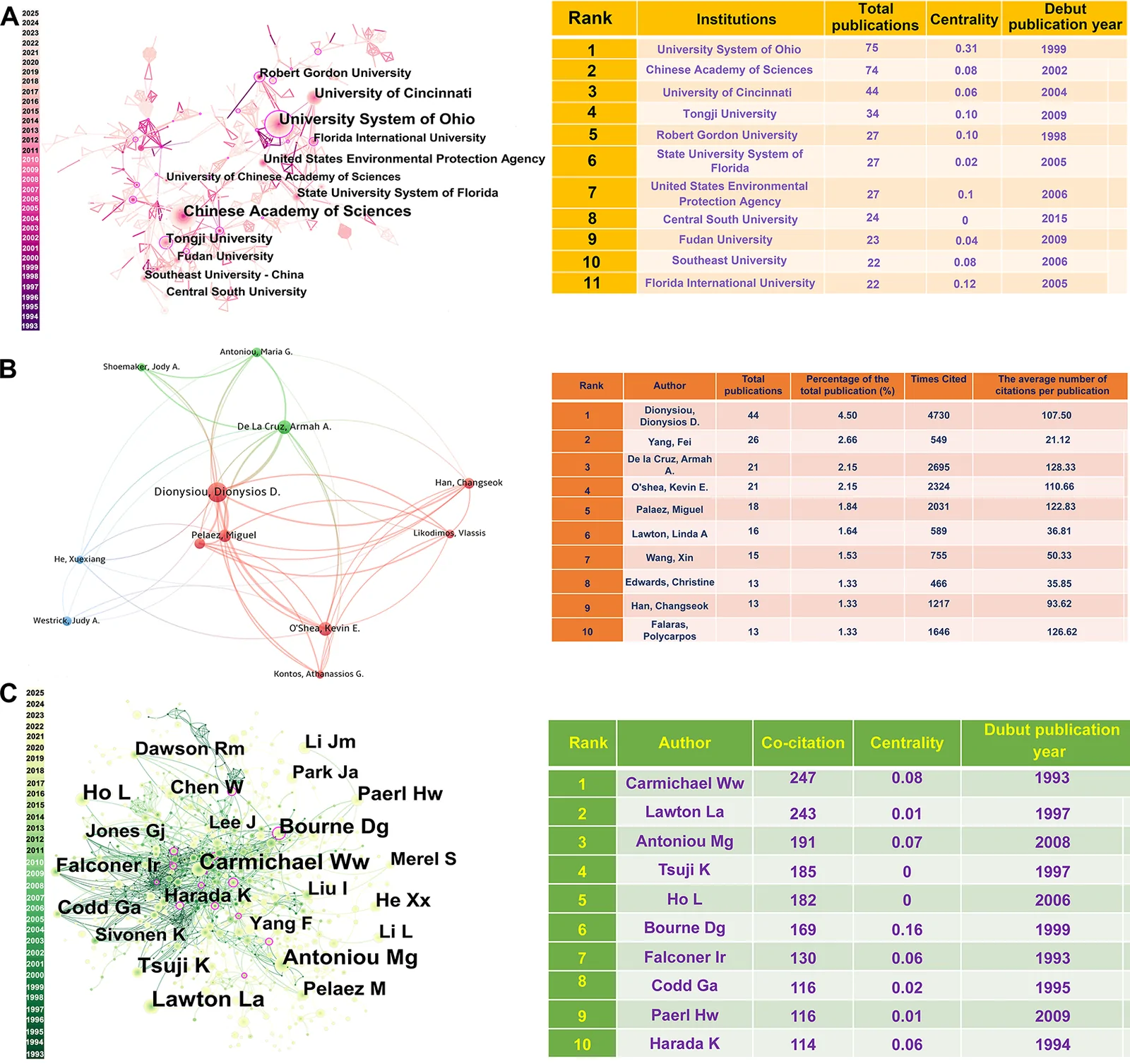
(A) Institutional contribution network for MC-LR removal research. Node size denotes publication volume, and annual ring width reflects yearly output. (B) Collaborative network of leading authors (eight or more publications). Node size corresponds to total output, and line thickness indicates collaboration strength. (C) Author co-citation network. Node size represents citation frequency, and annual ring width shows yearly citation activity.

Global distribution, institutional and author-level analyses further reveal a structured yet dynamically evolving collaboration network (figure 3). The institutional network comprises 731 nodes and 1291 links (density = 0.0048; supplementary material, Text S2), indicating a complex but cohesive research architecture. Institutions such as the University System of Ohio, Robert Gordon University and Tongji University exhibit high betweenness centrality (purple outer rings), serving as strategic bridges that facilitate knowledge exchange across distinct research clusters. The University System of Ohio anchors the network, maintaining strong partnerships with both domestic and international institutions, including Florida International University and the University of Chinese Academy of Sciences. Node size reflects publication volume, with the University System of Ohio leading (75 papers, 7.7%), followed by the Chinese Academy of Sciences (74, 7.6%), the University of Cincinnati (44, 4.5%) and Tongji University (34, 3.5%). Geographically, institutions in developed countries—particularly the United States—dominated early-stage research, while Chinese universities have recently assumed central positions, reflecting growing research capacity and global influence. Notably, Robert Gordon University (1998) and the University System of Ohio (1999) were among the earliest contributors, laying a foundation for subsequent advances in MC-LR removal.

At the individual level, 3694 authors have contributed to this field, forming three major collaborative clusters (figure 3B). The red cluster, centred on Dionysiou D.D., O'Shea K.E., Falaras P. and Pelaez M., represents the most active and interconnected collaboration network. The green cluster links De la Cruz A.A., Shoemaker J.A. and Antoniou M.G., while the blue cluster connects He X. and Westrick J.A. Dionysiou D.D. is the most prolific contributor (44 papers, 4.5%), followed by Yang F. (26) and De la Cruz A.A. (21). Notably, De la Cruz A.A. exhibits the highest academic influence (128.33 citations per paper), reflecting early and high-impact contributions to the field. The density map (supplementary material, fig. S3) reveals strong collaborative intensity around Dionysiou and O'Shea, whereas peripheral regions show weaker connections. The temporal evolution map (supplementary material, fig. S4) indicates that De la Cruz A.A. preceded Dionysiou D.D., marking an exploratory phase later expanded through Dionysiou's extensive output. Co-citation analysis (figure 3C; supplementary material, Text S3) identifies Carmichael W.W. (247 citations) and Lawton L.A. (243) as foundational scholars, with Carmichael W.W. showing the earliest debut year (1993) among the leading co-cited authors. The country-, institution- and author-level analyses reveal extensive international, interinstitutional, and interauthor collaboration in MC-LR removal research.

### 3.3. Analysis of keywords and co-cited references

**Figure 4.**
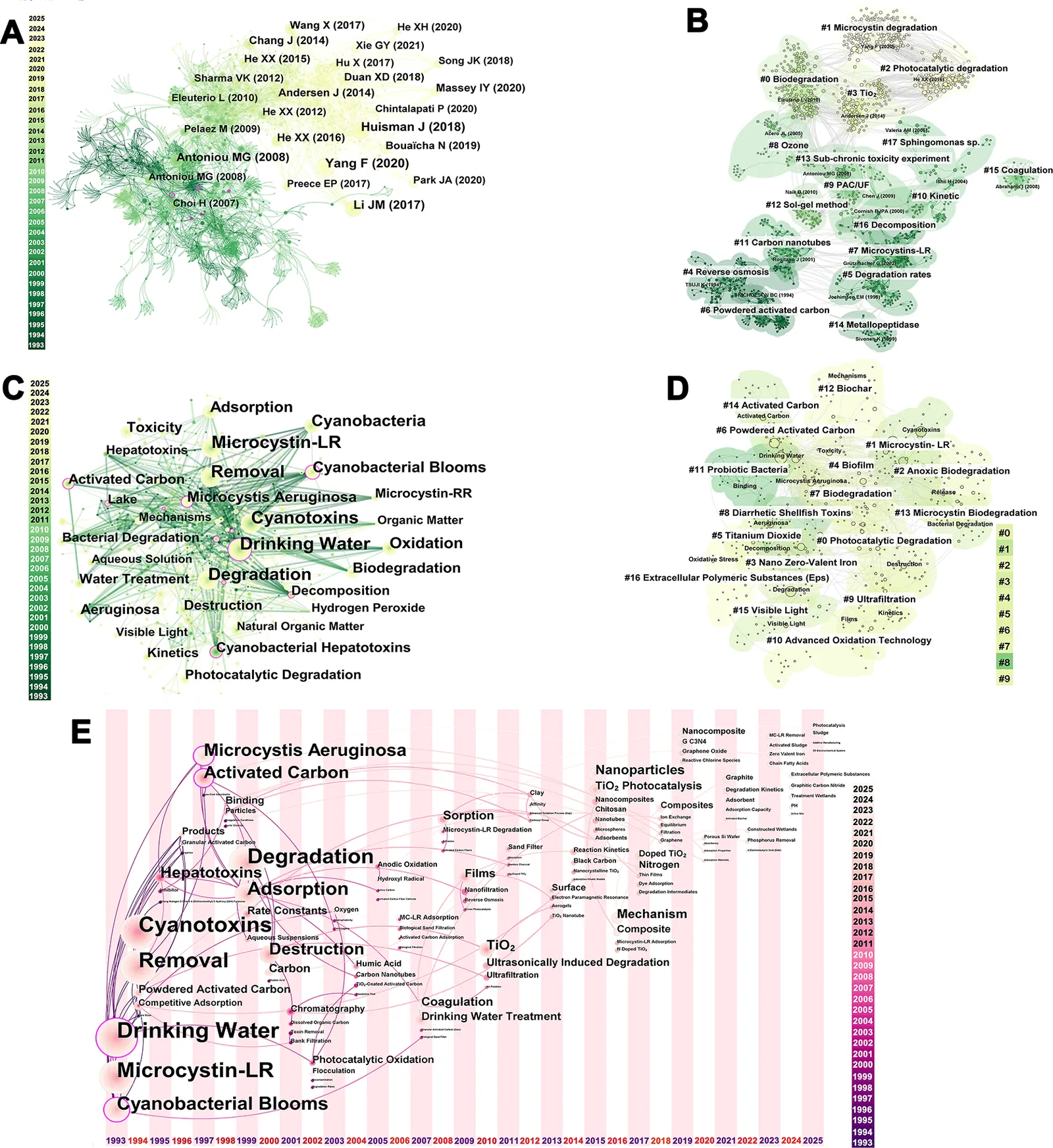
(A) Co-citation map showing major reference clusters and their interconnections. (B) Cluster structure of the MC-LR removal literature derived from co-citation patterns. (C) Keyword co-occurrence network, with node size proportional to co-occurrence frequency. (D) Keyword cluster map illustrating semantic groupings within the research domain. (E) Time-zone view of keywords showing the temporal emergence and evolution of research topics.

The co-citation analysis delineates the structural evolution of research on MC-LR removal (figure 4A,B, table 1; supplementary material, Text S4, Text S5 and table S3). The highly co-cited references in table 1 were grouped by document type. Li *et al.* [36], a review article, synthesized existing knowledge on microbial MC-LR degradation, whereas Yang *et al.* [5], an original research article, elucidated the complete microbial degradation pathway [5,36]. The co-citation network (1993–2025) comprises 870 highly co-cited references and demonstrates high structural reliability (*Q* = 0.8121; *S* = 0.9288). Cluster analysis identifies three major research directions. First, clusters #0 (‘Biodegradation’), #1 (‘Microcystin degradation’), #14 (‘Metallopeptidase’) and #17 (‘*Sphingomonas* sp.’) converge on microbial biodegradation mechanisms mediated by degrading bacteria and key enzymes. Second, clusters #2 (‘Photocatalytic degradation’), #3 (‘TiO₂’), #8 (‘Ozone’), #10 (‘Kinetic’), #11 (‘Carbon nanotubes’) and #12 (‘Sol–gel method’) highlight rapid advances in TiO₂- and carbon-based photocatalytic and oxidation processes. Third, clusters #4 (‘Reverse osmosis’), #6 (‘Powdered activated carbon’), #9 (‘PAC/UF’), #15 (‘Coagulation’) and #16 (‘Decomposition’) reflect increasing emphasis on multi-process treatment strategies integrating membrane separation, adsorption, coagulation and decomposition. These clusters map the field's progression from early conceptualization to diversified mechanistic and technological pathways for MC-LR removal.

**Table 1.** Documents with a co-citation frequency of no less than 23, grouped by document type.

first author	publication year	journal of publication	citation frequency	co-citation frequency	research content	reference
Yang, Fei	2020	*Water Research*	155	46	this study uncovers the complete mineralization pathway of MC-LR in a novel bacterium, Sphingopyxis sp. YF1, proving its potential for bioremediation.	[5]
Antoniou, Maria G.	2008	*Toxicon*	148	32	immobilized TiO₂ films on stainless steel degraded MC-LR in drinking water; neutral pH LC/MS detected 11 new intermediates, acidic conditions accelerated degradation, MS/MS revealed hydroxylation sites and fragmentation paths.	[30]
Andersen, Joel	2014	*Applied Catalysis B: Environment*	76	27	visible-light NF-TiO₂ photocatalysis degraded MC-LR via a likely hydroxyl-radical-mediated pathway, producing UV-like intermediates but with slower kinetics and reducing the toxin-associated structural moiety.	[31]
He, Xuexiang	2015	*Water Research*	103	23	V-254 nm processes (UV/S₂O₈²⁻ > UV/HSO₅⁻ > UV/H₂O₂) efficiently degraded MC-LR/RR/YR/LA via ·OH, outperforming cylindrospermopsin, with variable amino acids dictating kinetics and pathways.	[32]
Chang, Jing	2014	*Water Research*	93	26	ozonation of MC-LR yielded 7 intermediates (4 novel) and 6 aldehyde byproducts via Adda diene/Mdha attack; [O-3](o)/[MC-LR](o) >10 enabled peptide oxidation, dominating with formaldehyde, isovaleraldehyde, and methylglyoxal.	[33]
Wang, Xin	2017	*Applied Catalysis B: Environment*	110	23	NPT-EGC₄₅₀ achieved 99.4% MC-LR removal after 9 h, owing to enhanced photoabsorption and reduced charge-carrier recombination, with substantial mineralization to smaller products.	[34]
Duan, Xiaodi	2018	*Environmental Science & Technology*	95	23	UV/chlorine rapidly degraded MC-LR across pH 6—10.4, outperforming chlorination in reducing chlorinated byproducts and cytotoxicity (HepaRG viability 80%→90% at 96 mJ cm⁻²).	[35]
Li, Jieming	2017	*Science of the Total Environment*	232	39	to address MC-LR contamination, microbial degradation presents an efficient and eco-friendly solution.	[36]
He, Xuexiang	2016	*Harmful Algae*	310	26	multi-barrier strategy (prevention, source control, optimized treatment, monitoring), with a focus on evaluating the removal efficiency of cyanobacteria and toxins.	[37]
Massey, Isaac Yaw	2020	*Toxins*	130	24	bacterial biodegradation, a promising eco-friendly strategy for MC removal from water bodies, has garnered global attention.	[38]

*Note:* Publications 1–7 are original articles; publications 8–10 are reviews.

Analysis of the keyword co-occurrence network reveals a structured research landscape comprising 599 nodes and 1424 links (density = 0.008), where node size reflects co-occurrence frequency (figure 4C). The 10 most frequent keywords—*Drinking Water, Cyanotoxins, Microcystin-LR, Degradation, Removal, Cyanobacteria, Oxidation, Adsorption, Cyanobacterial Blooms* and *Microcystis aeruginosa*—indicate that current research primarily focuses on cyanobacterial bloom risks and the development of cyanotoxin mitigation technologies (supplementary material, table S4). The clustering map resolves 17 coherent keyword groups with high silhouette values (greater than 0.73), delineating two dominant thematic axes (figure 4D; supplementary material, Text S6; table S5). The first centres on materials and technology integration, highlighted by clusters such as *Titanium Dioxide, Nano Zero-Valent Iron, Biochar* and *Activated Carbon*, which align with high-frequency terms including *Photocatalytic Degradation, Visible Light, Advanced Oxidation Technology* and *Powdered Activated Carbon*. The second axis comprises biodegradation-oriented clusters, such as *Biodegradation, Microcystin Biodegradation* and *Anoxic Biodegradation*, reflecting the role of microbial pathways in transforming recalcitrant cyanotoxins. These patterns define the major research trajectories shaping MC-LR studies.

Temporal keyword mapping reveals changes in the relative prominence of research topics in MC-LR removal over time (figure 4E; supplementary material, fig. S5). Early work (1993–2005) is characterized by frequent occurrences of Activated Carbon, Decomposition, Adsorption, and Drinking Water, indicating an initial emphasis on adsorption-assisted removal and fundamental degradation processes. Between 2005 and 2015, terms such as Coagulation, Oxidation and Water Treatment Processes emerged, marking the introduction of chemical treatment and hybrid separation approaches. From 2017 onward, the keyword landscape shifts toward materials-enabled advanced oxidation, with TiO₂, Visible Light, Photocatalytic Oxidation, Nanoparticles and Composites becoming dominant. These patterns correspond to clusters including Photocatalytic Degradation (#0), Titanium Dioxide (#5) and Advanced Oxidation Technology (#10), highlighting intensified development of visible-light-responsive catalytic systems. In parallel, biodegradation-related clusters—Biodegradation (#7), Microcystin Biodegradation (#13) and Anoxic Biodegradation (#2)—indicate sustained exploration of microbial transformation pathways across both controlled and natural aquatic settings. Keyword burst analysis (supplementary material, fig. S6) further supports this progression, with early bursts in Activated Carbon giving way to recent bursts involving TiO₂ Photocatalysis, UV, Mechanisms and Reactive Oxygen Species. These temporal patterns reflect changes in the relative prominence of research topics rather than a linear succession of treatment approaches. Adsorption, oxidation and microorganism-mediated degradation represent overlapping research streams, while recent studies have placed greater emphasis on photocatalytic mechanisms, UV-based processes and reactive oxygen species.

## 4. Discussion

### 4.1. Annual output and core journals

Research on MC-LR removal has gradually evolved from an emerging issue into a continuously expanding, interdisciplinary field of study. The 2003 inflection (figure 1A) marks the shift from fragmented case studies to sustained research growth, driven by heightened awareness of cyanobacterial bloom risks, improved analytical capabilities and advances in water treatment technologies. In recent years, the annual publication output has stabilized at approximately 60 papers, indicating that publication activity in the field has entered a relatively stable phase following a period of rapid growth. However, this trend alone does not directly demonstrate that research methods have been standardized or that the relevant technologies have been translated into practical applications. This maturation is evident in the growing integration of mechanistic understanding with technological innovation. For instance, a nanobody–metal hybrid photocatalyst (Ag/AgCl/BSA-Nb) achieved nearly complete visible-light degradation of MC-LR with enhanced efficiency and reduced toxicity—demonstrating how molecular-level design can substantially improve treatment performance [39].

Visualization evidence further supports this trajectory. A concentrated share—368 publications (37.63%) across 10 high-impact environmental and engineering journals (figure 1B)—reflects a stable and methodologically coherent publication ecosystem [17]. Impact and citation patterns highlight a dual emphasis: technology-oriented studies in *Chemical Engineering Journal* and *Water Research*, mechanism-driven work in *Environmental Science & Technology* and complementary toxicological insight from *Toxicon*. Co-citation analysis (figure 1C) identifies *Water Research* and *Environmental Science & Technology* as intellectual hubs, linking environmental chemistry, materials engineering and toxicology, underscoring the increasingly interdisciplinary nature of the field. Although this study is subject to limitations related to database coverage, citation lag and reliance on journal-level evaluation metrics, publication trends, journal concentration, citation intensity and network centrality collectively indicate that MC-LR removal research has remained highly active and has developed relatively clear interdisciplinary knowledge linkages.

### 4.2. Global research landscape: leading countries, institutions and authors

The global distribution of MC-LR removal studies reveals distinct geographic and thematic structures (figure 2). China and the United States dominate publication output, jointly contributing more than two-thirds of total research. This concentration reflects China's policy-driven investment in water governance and expanding research capacity, alongside the United States’ sustained leadership in analytical methodology and technological innovation [17]. The international collaboration network shows an asymmetric but increasingly cohesive architecture, with the China–US partnership forming the global research axis with Australia, Canada and Japan acting as secondary hubs. Cite Space clustering further delineates seven thematic domains (#0–#6) tracing the field's transition from material innovation to applied environmental solutions (supplementary material, fig. S7). Early clusters, such as TiO₂ and Multi-Walled Carbon, underscore the dominance of photocatalytic and nanomaterial approaches, while later clusters emphasize ecological processes, degradation pathways and the toxicity of transformation products [40,41]. The emergence of advanced oxidation highlights a shift toward hybrid advanced oxidation processes (AOPs) validated under real-water conditions [42,43]. These spatial and thematic distribution patterns indicate that MC-LR removal research spans multiple areas, including materials science, environmental chemistry and toxicology, and has developed a collaborative structure centred on China–US cooperation with participation from other countries. Beyond China and the United States, countries such as India, Brazil, Greece and Poland have made distinctive contributions to MC-LR research.

Azevedo and colleagues in Brazil conducted an early study on toxic cyanobacteria and MC occurrence in the Utinga Reservoir, a major public drinking-water source, and examined MC concentrations in raw and treated water. Santos and colleagues reported MC-LR removal by small-sized bacterial communities using 16S rDNA sequencing and transmission electron microscopy [44], whereas Dziga's Polish group refined the Mlr enzymatic pathway through the characterization of MlrA, MlrB and MlrC [45,46]. Contributions from Greek researchers, including Kaloudis, and broader international collaborations further demonstrate the global nature of the field and show that scientific influence extends beyond publication volume [32].

Global structure and institutional-level patterns further demonstrate how research capacity is organized and channelled. Global research on MC-LR removal has transcended national boundaries, evolving into a multi-institutional and cross-disciplinary enterprise [17,47]. The institutional collaboration network reveals a dynamic yet organized structure anchored by the University System of Ohio, which serves as the primary hub connecting previously fragmented research communities. Although the Chinese Academy of Sciences (CAS) shows a lower betweenness centrality (0.08), it remains an important connector across several active clusters—particularly those focusing on mesoporous materials and advanced oxidation technologies [48]. These network properties indicate that scientific leadership depends less on publication volume than on centrality and knowledge brokerage. CiteSpace clustering of the institutional collaboration network identifies 20 institutional clusters labeled with representative research terms—from mesoporous materials and advanced oxidation technologies to TiO₂ photocatalysis [49,50]—underscoring the integration of materials innovation with mechanistic toxicology (supplementary material, fig. S8). Consistent with this integration, collaborative links among the University System of Ohio, Robert Gordon University and CAS exemplify important cross-institutional connections within an otherwise relatively sparse global network, while the growing roles of Fudan and Tongji Universities mark a geographic rebalancing toward emerging research economies.

MC-LR removal research organizes into three author clusters (figure 3B): a collaborative core around Dionysiou–O'Shea, a method-focused axis (De la Cruz–Shoemaker–Antoniou), and an implementation link (He, Westrick). Productivity and impact diverge—Dionysiou leads output, while De la Cruz achieves the highest per-paper influence, indicating concept-shaping early work. Density and temporal views (supplementary material, figs. S3 and S4) reveal a core–periphery structure and a shift from exploratory activity (2008–2010) to coordinated expansion, a pattern broadly consistent with previous bibliometric findings [47]. Co-citation patterns (figure 3C) further identify Carmichael and Lawton as foundational anchors connecting toxicology to treatment [51], with Carmichael's work representing one of the earliest contributions to the field in 1993. Complementing these actor-centric results, the author clustering map (supplementary material, fig. S9) delineates 20 thematic domains—with a consolidated core (#2 Biodegradation, #4 Advanced Oxidation, #3 TiO₂) surrounded by adsorption pathways (#6 Activated Carbon, #12 Adsorption, #7 PAC) and emerging fronts (#10 UV-LED, #9 Visible Light, #11 Ozone, #17 Biochar, #15 Microbial Fuel Cells)—this configuration indicates signalling convergence toward hybrid, systems-level solutions [49]. The network structure suggests that progress is organized by brokerage across clusters, not volume alone. Priorities are to strengthen interfaces (analytical chemistry ↔ AOP/photocatalysis ↔ deployment) and adopt shared benchmarks (matrices, MC-LR loads, transformation-product toxicity) to accelerate robust, scalable practice [52].

### 4.3. Analysis of keywords and co-cited references

The co-citation analysis shows a research field coalescing around a clearer mechanistic core and increasingly integrated technological strategies for MC-LR removal. The progression from the conceptual consolidation of Li *et al.* to the pathway-level resolution of Yang *et al.* reflects a decisive shift toward mechanistic maturity [5,36]. Three coherent domains now anchor the landscape. Early primary studies demonstrated the microbial degradation and biotransformation of MCs in aquatic environments further elucidated an enzymatic pathway for the bacterial degradation of MC-LR [10–13], while subsequent research has expanded the understanding of degrading microorganisms, enzymes and their potential application in biological treatment [18]. Early studies established TiO₂ photocatalysis as an effective approach for MC degradation and toxicity reduction [41,53,54], while subsequent research explored catalyst immobilization and more readily recoverable photocatalytic configurations [55,56]. Consistent with this evolution, the co-citation analysis shows that MC-LR removal research has diversified across conventional TiO₂ photocatalysis, UV-based advanced oxidation, ozonation and microbial degradation (figure 4A,B; table 1). These research directions indicate that microbial degradation, photocatalytic and oxidative treatment and multi-process treatment have emerged as relatively well-defined research themes. Further studies using real-water matrices and at the engineering scale are still needed to verify the stability and scalability of the relevant technologies.

The keyword co-occurrence network reveals a research field progressively organized around two converging trajectories. The dominance of terms such as Drinking Water, Cyanotoxins and MC-LR underscores a shift from bloom monitoring to targeted mitigation technologies. One trajectory focuses on materials- and technology-driven removal strategies, exemplified by photocatalytic systems (e.g. TiO₂) and advanced oxidation pathways [49]. The other trajectory highlights microbial biodegradation mechanisms, including both aerobic and anoxic processes [57]. These two axes now define the structural backbone of MC-LR research: advanced materials enabling rapid oxidative removal and biologically mediated systems offering transformation of persistent toxins. Rather than representing sequential stages, these approaches constitute two persistent and interrelated research directions. The keyword network indicates that material-driven treatment and microbial degradation are two persistent and interrelated research directions.

Temporal keyword evolution indicates the diversification of MC-LR research from foundational removal processes toward mechanism-driven and material-enabled strategies, rather than a strictly sequential transition. Early emphasis on Activated Carbon, Decomposition, and Adsorption reflects the field's initial reliance on adsorption-assisted pathways (1). Importantly, the exported keyword records indicate that ‘Bacterial Degradation’ and ‘Biodegradation’ first appeared in the network in 2001 and 2002, respectively. These dates refer to their first occurrence in the exported keyword network rather than the beginning of biological degradation research, which had already been represented by pioneering studies in the 1990s [10,11]. Overall, supplementary material, fig. S5 illustrates the overlapping development of biological and photocatalytic research directions rather than a strictly sequential progression. The emergence of *Coagulation* and *Oxidation* marks the adoption of broader chemical treatment concepts [18]. In recent years, the consolidation of terms such as TiO₂, Visible Light, Photocatalytic Oxidation and Nanoparticles highlight the rise of advanced, light-responsive catalytic systems [49,58], consistent with dominant clusters in the co-occurrence network. Parallel persistence of Biodegradation and MC Biodegradation indicates continued investment in microbial transformation pathways across environmental contexts [59]. The temporal keyword evolution and burst analysis indicate that MC-LR removal research has shifted from adsorption-assisted removal toward TiO₂-based photocatalysis, UV-based advanced oxidation and microbial degradation, thereby broadening practical treatment options from toxin separation to direct degradation.

These interpretations should be considered in light of several methodological caveats. Bibliometric patterns may be affected by database coverage, citation lag, keyword standardization, author and institution name disambiguation and algorithm-generated cluster labels. Experimental findings from natural waters, sediments, microbial cultures and enzyme-based systems are complementary but not directly comparable because treatment conditions and analytical endpoints differ. Moreover, disappearance of the parent MC-LR compound does not demonstrate complete detoxification unless transformation products and residual toxicity are assessed; therefore, laboratory-scale removal should not be equated with validated treatment performance in real-water or full-scale systems.

### 4.4. Challenges and future perspectives

Despite significant progress, the practical application of MC-LR removal technologies remains constrained by several challenges. First, future studies should move beyond apparent removal efficiency and incorporate detoxification efficiency, transformation-product identification and toxicity assessment as core indicators [43]. This is particularly important because adsorption mainly transfers MC-LR to the solid phase, whereas photocatalysis and advanced oxidation can degrade the toxin but may generate intermediate products with uncertain toxicity. Second, more studies should be conducted in real-water matrices, where dissolved organic matter, algal organic matter, inorganic ions, suspended particles and co-existing pollutants may interfere with adsorption sites, the generation of reactive oxygen species, catalytic activity and microbial degradation. Third, integrated treatment systems should be prioritized because single processes rarely achieve rapid removal, complete degradation, operational stability and economic feasibility simultaneously. Hybrid systems combining adsorption, membrane separation, advanced oxidation, photocatalysis and biodegradation may provide more robust solutions for cyanotoxin control. Fourth, future research should extend beyond MC-LR to other potentially toxic cyanobacterial non-ribosomal peptides, such as nodularins, anabaenopeptins and cyanopeptolins, with particular attention to their environmental occurrence, toxicity, transformation and responses to treatment processes. In addition, greater emphasis should be placed on their mixture effects under co-occurrence conditions, along with the development of integrated monitoring, risk assessment and coordinated removal technologies. Finally, standardized evaluation frameworks are needed to improve comparability among studies. Key parameters, including initial MC-LR concentration, water matrix characteristics, material dosage, reaction conditions, kinetics, transformation products, toxicity changes, regeneration performance, cost and scalability indicators, should be systematically reported [52]. These efforts will promote the transition of MC-LR removal research from laboratory-scale material optimization toward reliable, scalable and risk-oriented water management strategies.

## 5. Conclusion

This study systematically reveals the global research landscape, knowledge structure and technological evolution of MC-LR removal in aquatic environments over the past three decades. The results show that the field has gradually developed from scattered early explorations into a research area characterized by sustained growth in research output and an increasingly interdisciplinary nature. Since 1993, annual publication output has increased steadily, accelerated markedly after 2003 and stabilized at a relatively high level in recent years, indicating that publication activity in the field has entered a relatively stable phase following a period of rapid expansion. China and the United States dominate scientific output and constitute the central axis of international collaboration, while *Water Research, Chemical Engineering Journal* and *Environmental Science & Technology* serve as major platforms for knowledge dissemination and academic development. Keyword and co-citation analyses further show that physical adsorption, photocatalysis, advanced oxidation processes and biodegradation are the main technological pathways for MC-LR removal, with the field diversifying from an early emphasis on adsorption-assisted removal and conventional oxidation toward increasingly mechanism-driven, material-enabled and integrated strategies, while microbial degradation has remained an important parallel research direction. This diversification is reflected in the growing emphasis on TiO₂-based photocatalysis, visible-light-responsive systems, reactive oxygen species-mediated degradation and microbial transformation, highlighting a broader move from simple toxin removal to efficient degradation, toxicity reduction and system-level treatment design.

Despite these advances, practical application remains hindered by uncertain byproduct toxicity, limited validation and inconsistent evaluation. Future research should adopt standardized, risk-oriented frameworks that evaluate not only detoxification performance and potential toxicity, but also the feasibility of material regeneration and economic viability. In this context, treatment systems that combine physical adsorption using functional materials with complementary degradation or separation processes may offer more robust and scalable solutions for reliable cyanotoxin control.

## Supplementary Material



## Data Availability

The dataset used in this study consists of bibliographic records retrieved from the Web of Science database and exported as a savedrecs.txt file. The search strategy and literature screening criteria are detailed in §2.1. Based on this source file, CiteSpace and VOSviewer were used to extract author information and other data, which are provided in the supplementary material, Data Table folder. The extracted data files were then used as input data for generating the figures reported in the manuscript. No custom code was used in the data extraction, processing or figure generation. All relevant data files are openly available in the Open Science Framework repository [60]. Supplementary material is available online [61].
