# Supplementary material for: Three decades of microcystin-LR removal in aquatic environments: physical, chemical and biological strategies for environmental detoxification
Source: R Soc Open Sci. 2026 Sep 9;13(9):rsos260913. doi: 10.1098/rsos.260913 (PMC13561087; doi:10.1098/rsos.260913)
Supplement: Supplementary file 1 [file rsos.260913.zip › rsos.260913/rsos260913_si_001.docx]

Three decades of microcystin-LR removal in aquatic environments: physical, chemical and biological strategies for environmental detoxification

Xin Zhong^1^, Huixia Zhang*^1^, Ping Xie*^2^_’_^3^

*^1^ Longyan University, Longyan 364000, P.R. China.*

*^2^College of Life Sciences, Hebei University, Baoding 071002，P.R China*

*^3^Donghu Experimental Station of Lake Ecosystems, Institute of Hydrobiology, The Chinese Academy of Sciences, Wuhan 430072, P.R China*

_____________________

***Corresponding author**

E-mail address: lan642795613@163.com (Huixia. Zhang).

E-mail address: xieping@hbu.edu.cn (P. Xie).

**Table of contents**

**Page**

Fig. S1………………………………………………….……….............................S3

Table S1……………………………………….……………...................................S4

Table S2……………………………………….……………...................................S5

Text S1…………………………………………...…………...................................S6

Fig. S2………………………………………………….………..............................S7

Text S2…………………………………………...…………...................................S8

Fig. S3………………………………………………….………..............................S9

Fig. S4………………………………………………….………..............................S10

Text S3………………………………………………….………............................. S11

Text S4………………………………………………….………............................. S12

Text S5………………………………………………….………............................. S13

Table S3……………………………………….……………....................................S14

Table S4……………………………………….……………....................................S15

Text S6………………………………………………….………............................. S16

Table S5.……………………………………….……………...................................S17

Fig. S5………………………………………………….………..............................S18

Fig. S6………………………………………………….………..............................S19

Text S7………………………………………………….………............................ S20

Fig. S7………………………………………………….………..............................S21

Fig. S8………………………………………………….………..............................S22

Fig. S9………………………………………………….………..............................S23

References…………………………………………………….……..........................S24

**Fig. S1.** Study selection and analytic workflow for the bibliometric review of microcystin-LR removal.

**Table S1.** Keyword standardization applied before the bibliometric analysis.

| **Processing category** | **Original keyword(s)** | **Standardized keyword** | **Rationale** |
| --- | --- | --- | --- |
| Abbreviation standardization | rr | Microcystin-RR | Abbreviation expanded to the full congener name. |
| Abbreviation and spelling standardization | lr; mc lr; microcystin-lr (mc-lr) | Microcystin-LR | Abbreviations, capitalization, and hyphenation were standardized. |
| Abbreviation standardization | lr adsorption | MC-LR Adsorption | The abbreviated congener name was expanded and standardized. |
| Abbreviation standardization | lr removal | MC-LR Removal | The abbreviated congener name was expanded and standardized. |
| Terminology standardization | cyanobacterial toxins; cyanobacterial toxin; | Cyanotoxins | Singular and plural variants were consolidated under the standardized term. |
| Geographical-name standardization | lake taihu | Taihu Lake | Word order and capitalization were standardized. |
| Abbreviation and format standardization | reactive oxygen species (ros) | Reactive Oxygen Species | The parenthetical abbreviation and capitalization were standardized. |
| Chemical-notation standardization | tio2 | TiO₂ | Capitalization and subscript formatting were standardized. |
| Typographical correction | 001 active facets | Active Facets | An unintended numeric prefix was removed. |
| Typographical correction | 2 antibiotic contaminants | Antibiotic Contaminants | An unintended numeric prefix was removed. |

**Table S2** Top 10 journals by publication volume in MC-LR removal research.

| Journal | Total publications | Percentage of the total publications | Times cited | Average number of citations per publication |
| --- | --- | --- | --- | --- |
| Water Res. | 67 | 6.85 | 4676 | 69.79 |
| Chemosphere | 51 | 5.21 | 1684 | 33.02 |
| Chem. Eng. J. | 50 | 5.11 | 2177 | 43.54 |
| J. Hazard. Mater. | 42 | 4.29 | 1527 | 36.36 |
| Environ. Sci. Technol. | 36 | 3.68 | 3119 | 86.64 |
| Toxins | 28 | 2.86 | 316 | 11.29 |
| Sci. Total Environ. | 26 | 2.66 | 887 | 34.12 |
| Toxicon | 24 | 2.45 | 1388 | 57.83 |
| Environ. Pollut. | 23 | 2.35 | 783 | 34.04 |
| Sep. Purif. Technol. | 21 | 2.15 | 689 | 32.81 |

Note: Average citations per publication were calculated by dividing the total citations received by the MC-LR removal publications included in the present dataset by the number of included publications in each journal. This metric does not represent the journal-wide average citation rate across all subject areas.

**Text S1 Visualization of journal co-citation networks**

In the knowledge network generated by *CiteSpace*, each node represents a journal and encodes multiple attributes relevant to bibliometric interpretation, including journal identity, citation frequency, and co-citation connections. Node size scales with citation count—larger nodes signify higher scholarly influence and centrality, typically corresponding to core or foundational journals within the field. Color conveys temporal information through citation-year rings: cooler tones indicate earlier citation activity, reflecting journals that shaped the formative stages of MC-LR removal research, whereas warmer tones denote more recent activity, highlighting emerging venues and evolving research frontiers (Chen et al., 2021). The spatial distribution of nodes in the force-directed layout captures intellectual proximity—journals positioned closer together share greater thematic coherence and methodological overlap. Edges represent co-citation relationships, with line thickness indicating the strength of joint citation; thicker links reveal stronger intellectual coupling and tighter integration within the research structure (Chen, 2006; Chen et al., 2010). Collectively, these visual dimensions integrate influence, temporality, and connectivity, offering an interpretable map of how journals interact, evolve, and organize the intellectual architecture of MC-LR removal research.

**Fig. S2.** This figure shows the international collaboration network among the top five countries in MC-LR removal research (≥44 publications per country). Node size reflects total publication output, and link thickness represents the strength of bilateral collaboration.

**Text S2. Collaborative network among institutions**

**In the institutional collaboration network, each node represents a research institution, with node size and label indicating total publication output—larger nodes signify higher productivity. The concentric growth rings reflect annual publication activity, where ring width corresponds to the number of papers produced in a given year. Nodes outlined in purple denote high betweenness centrality, highlighting institutions that bridge distinct research clusters and act as key intermediaries in knowledge exchange. Links between nodes represent collaborative relationships, with line thickness proportional to the strength of cooperation and line color indicating the year in which the partnership was established (Chen et al., 2024; Jia and Harji, 2023).**

**Fig. S3.** Density map visualizing author collaboration in MC-LR removal studies. Darker colors indicate higher frequencies of co-authorship. Warm colors (e.g., red, yellow) represent the core of the most intensive and active author collaboration, while cool colors (e.g., blue, green) indicate the periphery with less frequent cooperation.

**Fig. S4.** Network visualization analysis of author collaboration in MC-LR removal studies. Colors represent the time of collaboration.

**Text S3. Author co-citation network**

In the knowledge graph generated by CiteSpace, each node serves as an analytical unit containing multidimensional information. The size of the node intuitively reflects the scholar's citation frequency, with larger nodes indicating greater influence and core status in the field, typically corresponding to foundational figures or proposers of key theories. The node's color, particularly its outer “citation year rings,” carries crucial temporal information: cool tones (such as blue and green) denote widespread citations of the scholar's work in the early stages, likely representing pioneers in the field; whereas warm tones (such as yellow and red) signify recent citation activity, potentially highlighting emerging research trends. The position of nodes within the network reveals academic affinities, with scholars located closer together exhibiting higher similarity in research themes, often belonging to the same subfield or sharing comparable theories and methodologies. Finally, the links between nodes symbolize co-citation relationships, where the thickness of the link directly represents the frequency with which two scholars are jointly cited by subsequent studies; thicker links indicate stronger academic connections.

**Text S4. Co-citation network of literature**

In a co-citation network generated by Cite Space, nodes represent cited references, with their size proportional to the citation frequency, thereby serving as an indicator of a reference's influence. The “tree-ring”structure of each node further illustrates the temporal distribution of its influence. Links between nodes denote co-citation relationships, and the thickness of these links corresponds to the co-citation strength, reflecting the degree of association between two references in subsequent research. Color serves two primary functions: first, to represent temporal progression, where a color gradient from cool (e.g., forest green) to warm hues (e.g., lime and yellow) visualizes the evolution from earlier to more recent research, aiding in the identification of foundational knowledge and emerging frontiers; second, to distinguish research clusters, whereby nodes of the same color form a thematically coherent subgroup. Together, these elements—node size and structure, link thickness, and color coding—provide an intuitive visualization of a field's intellectual structure, developmental trajectory, and research fronts.

**Text S5. Identification and validation of research clusters**

Clusters represent groups of nodes that are tightly interconnected through semantic or citation relationships, typically corresponding to distinct research themes or emerging fronts within a broader scientific domain. The size of a cluster reflects the relative prominence or intensity of research activity in that area; larger clusters usually indicate greater scholarly attention and thematic concentration. In knowledge-mapping software such as CiteSpace, clusters are commonly identified and labeled using the log-likelihood ratio (LLR) algorithm. Cluster identifiers are numbered sequentially (0, 1, 2, …), with smaller indices denoting larger clusters that encompass more keywords. Each cluster comprises multiple terms that are semantically or co-occurrence related, and the detailed composition of each cluster can be obtained from the analytical output. The structural quality of the clustering is assessed primarily using two metrics. The modularity index (Q) measures the overall significance and degree of separation among clusters; values of Q > 0.3 generally indicate a statistically meaningful structure. The silhouette coefficient (S) evaluates the internal consistency and external distinctiveness of clusters; values of S > 0.5 suggest acceptable structural reliability, whereas S > 0.7 denote highly robust and credible clustering results.

**Table S3** Detailed parameters of literature clusters

| Cluster ID | Cluster Size | Silhouette Value | Average Year |
| --- | --- | --- | --- |
| 0 | 192 | 0.794 | 2011 |
| 1 | 178 | 0.909 | 2020 |
| 2 | 145 | 0.877 | 2017 |
| 3 | 133 | 0.913 | 2013 |
| 4 | 114 | 0.96 | 1993 |
| 5 | 108 | 0.928 | 1998 |
| 6 | 92 | 0.961 | 1993 |
| 7 | 75 | 0.947 | 2001 |
| 8 | 75 | 0.99 | 2006 |
| 9 | 72 | 0.909 | 2004 |
| 10 | 68 | 0.972 | 2002 |
| 11 | 68 | 0.961 | 2000 |
| 12 | 62 | 0.995 | 2007 |
| 13 | 45 | 0.983 | 2004 |
| 14 | 37 | 0.988 | 1997 |
| 15 | 35 | 1 | 2006 |
| 16 | 33 | 0.979 | 1997 |
| 17 | 32 | 0.965 | 2003 |

**Table S4** Only retain keywords with co-citation frequency ≥100.

| Rank | Co-citation frequency | Centrality | Publication year | Keywords |
| --- | --- | --- | --- | --- |
| 1 | 326 | 0.11 | 1993 | Drinking Water |
| 2 | 286 | 0.03 | 1994 | Cyanotoxins |
| 3 | 271 | 0.04 | 1993 | Microcystin-LR |
| 4 | 257 | 0.09 | 1999 | Degradation |
| 5 | 252 | 0.03 | 1994 | Removal |
| 6 | 181 | 0.06 | 1993 | Cyanobacteria |
| 7 | 135 | 0.01 | 1998 | Oxidation |
| 8 | 130 | 0.09 | 1999 | Adsorption |
| 9 | 127 | 0.16 | 1993 | Cyanobacterial Blooms |
| 10 | 101 | 0.15 | 1997 | Microcystis Aeruginosa |

**Text S6 Keyword clusters: Green labels**

The figure contains 17 green labels, each representing a cluster. Each cluster's label is a keyword from the co-occurrence network. A larger cluster ID number indicates fewer keywords in the cluster, while a smaller cluster ID number indicates more keywords. Connections between nodes within a cluster represent co-occurrence relationships. A higher number of connections suggests stronger co-occurrence among the cluster's keywords, reflecting a more cohesive research theme in that area.

**Table S5** Detailed parameters of keyword clusters

| Cluster ID | Cluster Size | Silhouette Value | Average Year |
| --- | --- | --- | --- |
| 0 | 54 | 0.853 | 2013 |
| 1 | 48 | 0.849 | 2002 |
| 2 | 47 | 0.779 | 2016 |
| 3 | 41 | 0.86 | 2008 |
| 4 | 41 | 0.824 | 2009 |
| 5 | 38 | 0.862 | 2011 |
| 6 | 35 | 0.876 | 2003 |
| 7 | 34 | 0.907 | 2011 |
| 8 | 32 | 0.879 | 2000 |
| 9 | 32 | 0.738 | 2015 |
| 10 | 28 | 0.952 | 2018 |
| 11 | 25 | 0.91 | 2004 |
| 12 | 24 | 0.856 | 2015 |
| 13 | 24 | 0.951 | 2006 |
| 14 | 23 | 0.935 | 2007 |
| 15 | 21 | 0.876 | 2012 |
| 16 | 17 | 0.984 | 2012 |

**Fig. S5.** Evolution of the research theme on MC-LR removal. The Y-axis denotes keyword clusters representing research themes, while the X-axis indicates publication year. Node size reflects the intensity of keyword bursts, and the connecting lines illustrate their co-occurrence relationships.

**Fig. S6.** The top 25 keywords exhibiting the most significant citation surges in MC-LR removal research

**Text S7 Top citation burst keywords**

In **Fig. S6**, the top 25 keywords exhibiting the strongest citation bursts in MC-LR removal research are presented. The blue line represents the timeline, while the red line indicates the duration of the citation burst, illustrating the evolution of trending topics. The Strength value in **Fig. S6** reflects the level of attention a topic received; higher values denote more prominent keywords. The "Year" in the second column indicates the first appearance of the keyword. In the last column, the blue bar spans from January 1993 to August 2025, whereas the red bar marks the period of burst and peak attention for the keyword.

**Fig. S7.** The CiteSpace-based map visualizes the collaboration and thematic clustering among major countries involved in MC-LR removal research from 1993 to 2025.

**Fig. S8.** Clusters of the global institutional collaboration network in MC-LR removal research.

**Fig. S9.** Global author clusters in MC-LR removal research

**References**

Chen, C.M. 2006. CiteSpace II: Detecting and visualizing emerging trends and transient patterns in scientific literature. J. Am. Soc. Inf. Sci. Technol. 57(3), 359-377 <Https://.doi.org/10.1002/asi.20317>.

Chen, C.M., Ibekwe-SanJuan, F. and Hou, J.H. 2010. The structure and dynamics of cocitation clusters: A multiple-perspective cocitation analysis. J. Am. Soc. Inf. Sci. Technol. 61(7), 1386-1409 <Https://.doi.org/10.1002/asi.21309>.

Chen, X., Cheng, X., Meng, H., Selvaraj, K.K., Li, H., He, H., Du, W., Yang, S., Li, S. and Zhang, L. 2021. Past, present, and future perspectives on the assessment of bioavailability/bioaccessibility of polycyclic aromatic hydrocarbons: A 20-year systemic review based on scientific econometrics. Sci. Total Environ. 774, 145585 <Https://.doi.org/10.1016/j.scitotenv.2021.145585>.

Chen, X.N., Chen, X. and Lai, Y. 2024. Development and emerging trends of drug resistance mutations in HIV: a bibliometric analysis based on CiteSpace. Front. Microbiol. 15 <Https://.doi.org/10.3389/fmicb.2024.1374582>.

Jia, S.J. and Harji, M.B. 2023. Themes, knowledge evolution, and emerging trends in task-based teaching and learning: A scientometric analysis in CiteSpace. Educ. Inf. Technol. 28(8), 9783-9802 <Https://.doi.org/10.1007/s10639-023-11586-y>.
